# Successful Treatment of Infective Endocarditis With Oral Antibiotics: A Case Report

**DOI:** 10.7759/cureus.43514

**Published:** 2023-08-15

**Authors:** Ali Alsaeed, Mousa J Alhaddad, Abdullah A AlKhalaf, Ashraf Alkhudair, Naif Alqannas

**Affiliations:** 1 Infectious Disease, Dammam Medical Complex, Dammam, SAU; 2 Internal Medicine, Dammam Medical Complex, Dammam, SAU; 3 Saud Albabtain Cardiac Center, Dammam Medical Complex, Dammam, SAU

**Keywords:** echocardiogram, saudi arabia, rifampin, rifampicin, linezolid, oral antibiotics, staphylococcus aureus bacteremia, methicillin-sensitive staphylococcus aureus, tricuspid valve endocarditis, infective endocarditis

## Abstract

Infective endocarditis (IE) is a serious and potentially life-threatening infection of the heart valves. It is commonly treated with prolonged courses of intravenous antibiotics, and in some cases, surgical intervention may also be necessary. While the use of oral antibiotics in the treatment of IE is generally limited, there are select cases where they may be considered as an alternative treatment option. Here, we report a case of staphylococcal right-sided IE successfully treated with oral antibiotics (linezolid and rifampicin). Our case highlights the potential for oral antibiotics to be used as step-down therapy for select patients with IE.

## Introduction

Infective endocarditis (IE) is a frighting disease with a global age-standardized incidence rate (ASIR) of 13.80 per 100,000 person-years [1]. Its incidence in Saudi Arabia is 15 cases per 100,000 hospital admissions [2,3]. Despite advances in medical care and the availability of potent antimicrobial agents, it remains a challenging condition to manage, with high morbidity and mortality rates [4-8]. The optimal management of IE involves a multidisciplinary approach, including infectious disease specialists, cardiologists, and cardiothoracic surgeons, and typically requires prolonged courses of intravenous antibiotics. In some cases, surgical intervention may also be necessary [9,10], and it is performed in 25-27% of IE patients in Saudi Arabia [6-8].

Intravenous antibiotics are the standard of care for the treatment of IE [9-11], as oral antibiotics may not achieve therapeutic levels in the bloodstream or heart tissue, which can result in treatment failure or relapse. However, oral antibiotics have been used as a step-down therapy in some cases of IE, particularly in patients who are clinically stable and have no evidence of systemic infection or endocarditis-related complications, and in cases where the infecting organism is susceptible to oral antibiotics and close monitoring and follow-up are ensured [12,13]. This approach was supported by the Partial Oral Treatment of Endocarditis (POET) trial [14]. However, the trial did not include patients with right-sided endocarditis. In addition, it excluded patients who had signs of abscess formation or valve abnormalities on transesophageal echocardiography (TEE) before randomization [14,15].

Here, we present the case of a 17-year-old male patient with no significant medical history who was diagnosed with right-sided IE caused by *Staphylococcus aureus*. The patient, who has persistent vegetation on TEE, was treated initially with intravenous antibiotics but was later discharged on oral antibiotics due to financial constraints. Despite the use of oral antibiotics, which is not a common practice for treating infective endocarditis in Saudi Arabia, the patient showed clinical improvement and had no evidence of relapse during follow-up.

## Case presentation

The patient was a 17-year-old male of Pakistani origin, born and raised in Saudi Arabia, with no legal residency status and no significant past medical history. He presented to a private hospital with constitutional symptoms of fever, chills, decreased appetite, and shortness of breath for two months. Blood cultures were positive for *S. aureus*, and he was suspected to have IE. The patient was treated with intravenous antibiotics (cefazolin and gentamicin) for around seven days and then discharged on oral clindamycin to be evaluated at a higher center.

**Figure 1:**
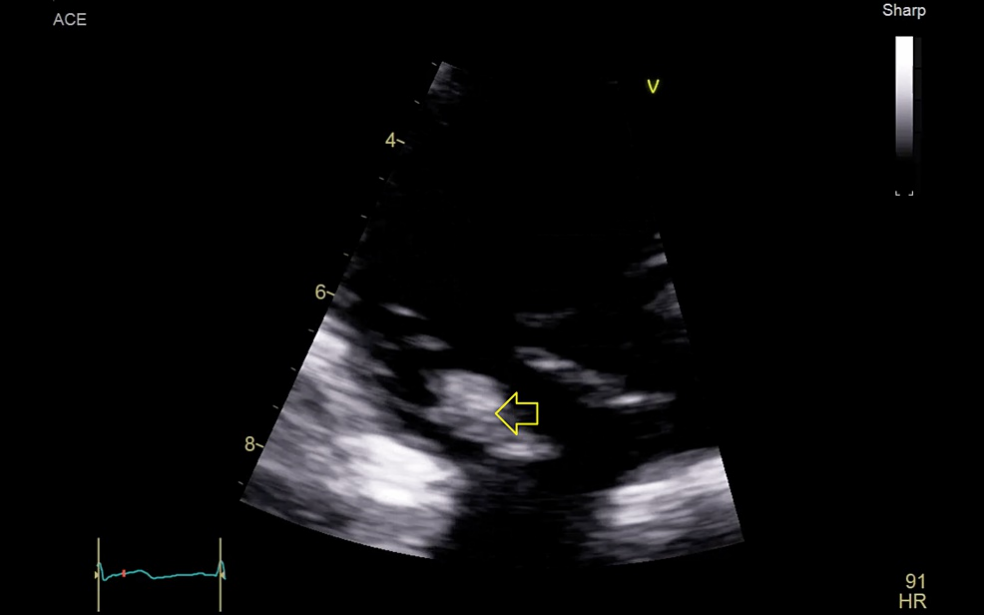
An image of the tricuspid valve of the first transthoracic echocardiogram showing a large mass (arrow) attached to a tricuspid valve leaflet.

**Figure 2:**
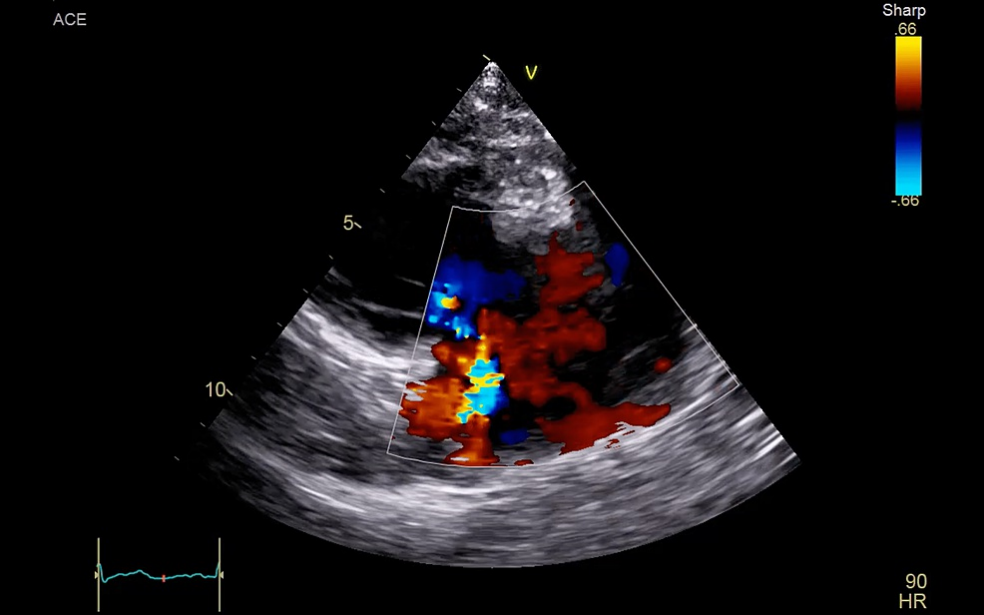
A right ventricular inflow view with color doppler of the first transthoracic echocardiogram showing tricuspid regurgitation.

Ten days later, the patient presented to Dammam Medical Complex seeking further management (day 0). Blood cultures were sent. A chest computed tomography (CT) scan revealed pulmonary septic emboli, and transthoracic echocardiography (TTE) showed vegetation on the tricuspid valve with severe tricuspid regurgitation (Figures 1-2).

**Figure 3:**
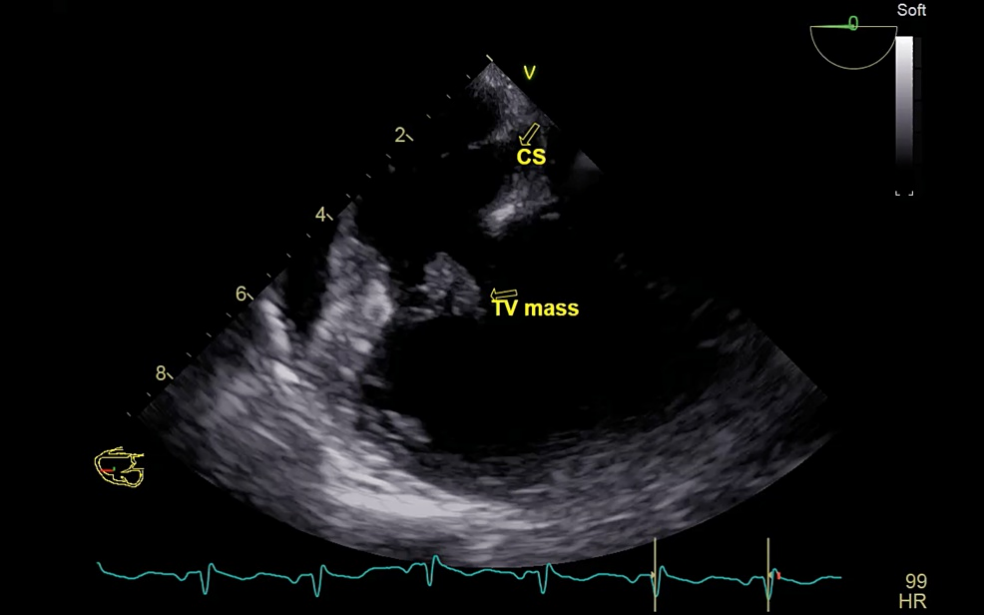
A lower esophageal view at 0 degree of the first transesophageal echocardiogram showing coronary sinus (CS) and a large mass attached to posterior leaflet of the tricuspid valve (TV).

**Figure 4:**
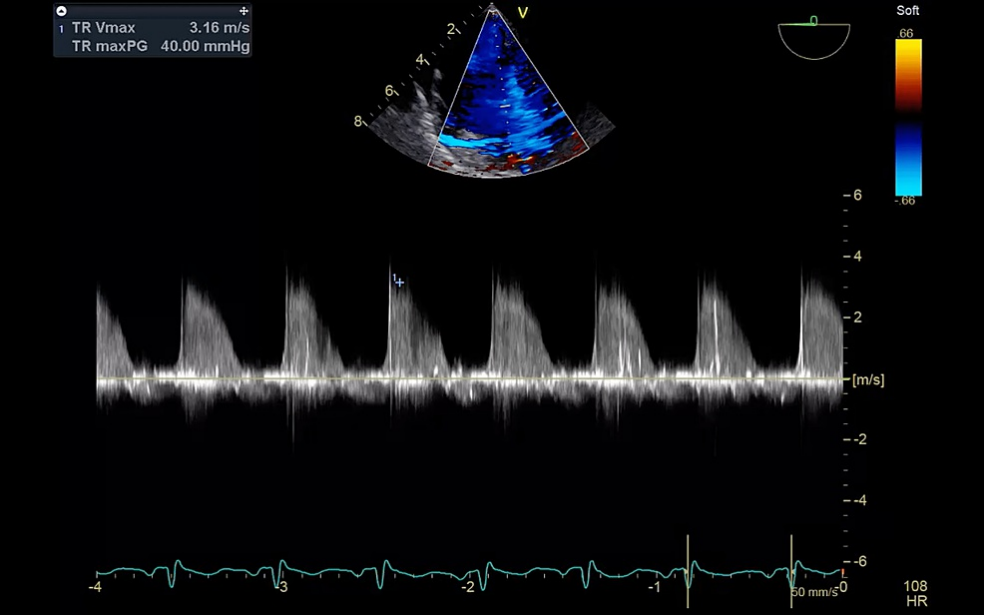
A continuous wave doppler showing early peaking signal which is suggestive of severe tricuspid regurgitation.

**Figure 5:**
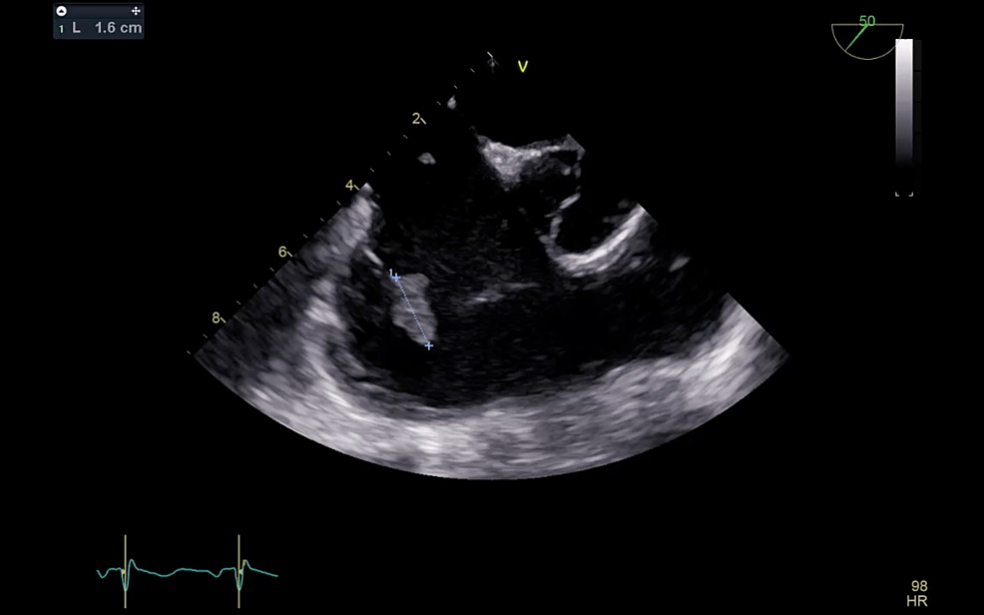
A mid-esophageal short access view of the first transesophageal echocardiogram at the level of the aortic valve showing a large mass (dashed line) attached to the anterior and posterior tricuspid valve leaflets.

The vegetation in the tricuspid valve was confirmed by TEE with ruptured chordae and a flail leaflet (Figures 3-5).

The patient was initially treated with intravenous vancomycin and cefazolin, but after the blood cultures showed methicillin-susceptible *S. aureus* (MSSA), vancomycin was discontinued (day 6). The cardiology team assessed the patient and monitored him with a plan for surgical intervention in case he failed to respond to antibiotic therapy. Of note, the patient was not eligible for outpatient parenteral antimicrobial therapy (OPAT) due to his illegal residency status and because the home care team deemed it difficult to administer cefazolin every eight hours at home.

Later, the family opted to leave against medical advice after a family meeting due to financial constraints (day 17), and the patient was discharged on oral linezolid (600 mg twice daily) and rifampicin (300 mg twice daily). He was hemodynamically stable upon discharge. However, his bacteremia had not cleared yet. He was given weekly follow-up appointments for repeating blood investigations, including complete blood counts, renal function tests, liver function tests, and blood cultures, with a plan for readmission if he shows any signs of failure of therapy (e.g., breakthrough fever, leukocytosis, and persistent bacteremia).

The patient presented to the outpatient department two weeks after discharge, and all blood cultures showed no growth (day 33). No adverse effects from the antibiotics were reported. However, a TTE done four weeks later (day 60) revealed persistent vegetation (2.3 cm × 0.8 cm).

Finally, the cardiology team agreed to proceed with surgical intervention for the persistent large vegetation, but due to financial limitations, it was not performed. There was an agreement, though, to pursue surgery if the blood cultures regained positivity. Thus, the antibiotics were stopped (day 68). Fortunately, the follow-up blood cultures were negative. A follow-up TEE revealed that the tricuspid vegetation had disappeared with a residual thickness on the posterior tricuspid valve leaflet (day 111).

**Figure 6:**
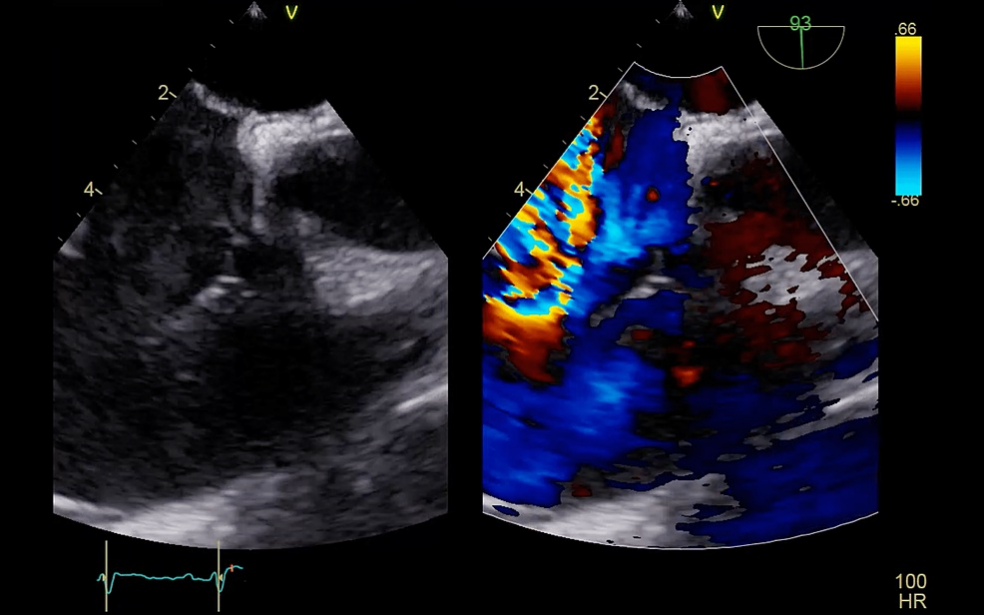
A modified bicaval view with color comparison of the last transesophageal echocardiogram showing a flail anterior TV leaflet with severe TR and no vegetation.

Six months later, the patient underwent repeat blood cultures, chest CT, and abdominal CT, which were all negative for infection. A TEE was also done, revealing no vegetation, but there was persistent severe tricuspid valve regurgitation with a destroyed, failed leaflet (Figure 6).

## Discussion

The management of IE typically involves the use of prolonged courses of intravenous antibiotics [13]. However, the use of these antibiotics carries several disadvantages, including the need for hospitalization, the risk of complications such as catheter-related infections, and the high cost of treatment [12,16]. In addition, the use of intravenous antibiotics may limit the patient's mobility and quality of life [17].

In this case report, the patient was diagnosed with IE caused by MSSA based on two major modified Duke criteria (positive blood cultures and evidence of endocardial involvement), in addition to the presence of two minor criteria (fever and septic pulmonary emboli). He was initially treated with intravenous antibiotics for two weeks. However, he was later switched to oral linezolid and rifampicin due to financial constraints.

Linezolid is a synthetic oxazolidinone antibiotic that is active against a wide range of Gram-positive organisms [18,19]. Due to its 100% bioavailability for its oral route [18], linezolid is considered a favorable oral option for IE treatment. However, the fact that it is bacteriostatic has limited its use in IE [20], an infection that was classically and traditionally treated with bactericidal antibiotics [11]. Nevertheless, linezolid was shown to be effective for the treatment of MSSA, methicillin-resistant *S. aureus* (MRSA), and vancomycin-intermediate *S. aureus* (VISA) endocarditis [21-29]. It was also shown to be effective for the treatment of endocarditis caused by streptococci and enterococci [27,28,30]. Patients needing prolonged use of linezolid (e.g., patients with IE) should be monitored for thrombocytopenia since it can develop in 31% of patients [25]. They also need to be monitored for peripheral neuropathy and optic neuritis, which may not be reversible [11]. Their medication should also be screened for any drug that could interact with linezolid (e.g., selective serotonin reuptake inhibitors) [11].

Rifampicin, on the other hand, is a rifamycin antibiotic that has excellent penetration into cardiac tissue. It is often combined with other antibiotics to provide a synergistic effect [21,31-36]. However, it is linked to the emergence of resistant *S. aureus* isolates [37-39]. To decrease this resistance, rifampicin is usually delayed for several days to allow other anti-staphylococcal antibiotics to achieve adequate penetration into the cardiac vegetation and decrease the bacterial burden [9,11]. Rifampicin can also lead to significant drug-drug interactions [37], and cautions should be taken for its use in patients with mechanical heart valves on oral anticoagulants using vitamin K antagonists when they are suspected to have IE as it might normalize the international normalized ratio (INR) [40,41]. It was also linked to hepatotoxicity [37], but the hepatotoxicity that is associated with rifampicin use may be less than that associated with the intravenous antibiotics used for IE (e.g., oxacillin) [42]. It must be noted that rifampicin is only recommended as an adjunctive antibiotic to other intravenous antibiotics for *S. endocarditis* on prosthetic valves since adding it to the treatment of *S. bacteremia* or native valve endocarditis has not been shown to be beneficial [9,11,37,43-46]. It remains unclear, however, whether combining rifampicin in an oral regimen for IE will be of any additive value.

Although the effectiveness of oral antibiotics (such as ciprofloxacin and rifampicin) in treating patients with right-sided S. endocarditis might have been evident in the 1990s [42,47,48], it was not until the results of the POET trial were published that the popularity of oral antibiotics for IE increased [13,15]. The POET trial is a recent randomized, non-inferiority, multicenter trial that evaluated the efficacy and safety of switching from intravenous to oral antibiotics in patients with stable endocarditis on the left side of the heart caused by Streptococci, *Enterococcus faecalis*, *S. aureus*, or coagulase-negative staphylococci. The study included 400 adults who were initially treated with intravenous antibiotics and were then randomized to continue intravenous treatment or switch to oral antibiotic treatment. It concluded that changing to oral antibiotic treatment was as effective and safe as continued intravenous antibiotic treatment [14]. However, the results of this trial are not generalizable to all IE patients yet, as around 80% of the patients who were assessed for trial eligibility were excluded. Examples of the excluded patients include patients who had a high level of white blood cells or C-reactive protein, patients with an impaired immune response, and patients with suspected reduced gastrointestinal uptake. In addition, all the patients were assessed by TEE before randomization to ensure that they did not have signs of abscess formation or valve abnormalities that would require surgery [14]. Of note, the combination of linezolid and rifampicin was among the recommended oral regimens in the POET trial for MSSA, along with dicloxacillin/fusidic acid, dicloxacillin/rifampicin, and linezolid/fusidic acid [14]. However, the dose of rifampicin used at 600 mg twice daily was higher than the dose given to the presented patient in this case report.

## Conclusions

The case report added to the growing evidence supporting the efficacy of oral antibiotics as a step-down therapy in selected patients with IE, particularly those without comorbidities who are clinically stable and overall improving, can tolerate oral medications, have normal absorptive gastrointestinal function, and have no signs of abscess formation. However, careful patient selection and close monitoring are essential to ensuring treatment success.
